# Supplementary material for: Vector distribution and transmission risk of the Zika virus in South and Central America
Source: PeerJ. 2019 Nov 7;7:e7920. doi: 10.7717/peerj.7920 (PMC6863140; doi:10.7717/peerj.7920)
Supplement: Supplemental Information 1 [file peerj-07-7920-s001.docx]

**Table S1:** Links (url) to occurrence data and predictor variables used for ecological niche modelling.

| Data | url |
| --- | --- |
| Occurrence data for the vector species | http://dx.doi.org/10.5061/dryad.47v3c |
| Climate data | www.worldclim.org |
| Land cover data | http://due.esrin.esa.int/page_globcover.php |
| Human Zika virus occurrence | https://doi.org/10.6084/m9.figshare.2573629.v1 |
| Temperature of warmest quarter | www.worldclim.org |
| Zika evidence consensus map | Compiled according to Fig. 2,  provided as supplementary asci file |
| Dengue evidence consensus map | https:// doi.org/10.1371/journal.pntd.0001760 |
| Gross domestic product per capita | https://data.worldbank.org/ |
| Population density | https://www.gideononline.com/, https://www.worldatlas.com |

**Table S2:** GlobeCover classes

| Code | GlobeCover 2009 |
| --- | --- |
| 14 | Rainfed croplands |
| 30 | Mosaic vegetation (grassland/shrubland/forest) (50-70%) / cropland (20-50%) |
| 40 | Closed to open (>15%) broadleaved evergreen or semi-deciduous forest (>5m) |
| 50 | Closed (>40%) broadleaved deciduous forest (>5m) |
| 60 | Open (15-40%) broadleaved deciduous forest/woodland (>5m) |
| 70 | Closed (>40%) needleleaved evergreen forest (>5m) |
| 90 | Open (15-40%) needleleaved deciduous or evergreen forest (>5m) |
| 100 | Closed to open (>15%) mixed broadleaved and needleleaved forest (>5m) |
| 110 | Mosaic forest or shrubland (50-70%) / grassland (20-50%) |
| 120 | Mosaic grassland (50-70%) / forest or shrubland (20-50%) |
| 130 | Closed to open (>15%) (broadleaved or needleleaved, evergreen or deciduous) shrubland (<5m) |
| 140 | Closed to open (>15%) herbaceous vegetation (grassland, savannas or lichens/mosses) |
| 150 | Sparse (<15%) vegetation |
| 160 | Closed to open (>15%) broadleaved forest regularly flooded (semi-permanently or temporarily) - Fresh or brackish water |
| 170 | Closed (>40%) broadleaved forest or shrubland permanently flooded - Saline or brackish water |
| 180 | Closed to open (>15%) grassland or woody vegetation on regularly flooded or waterlogged soil - Fresh, brackish or saline water |
| 190 | Artificial surfaces and associated areas (Urban areas >50%) |
| 200 | Bare areas |
| 210 | Water bodies |
| 220 | Permanent snow and ice |


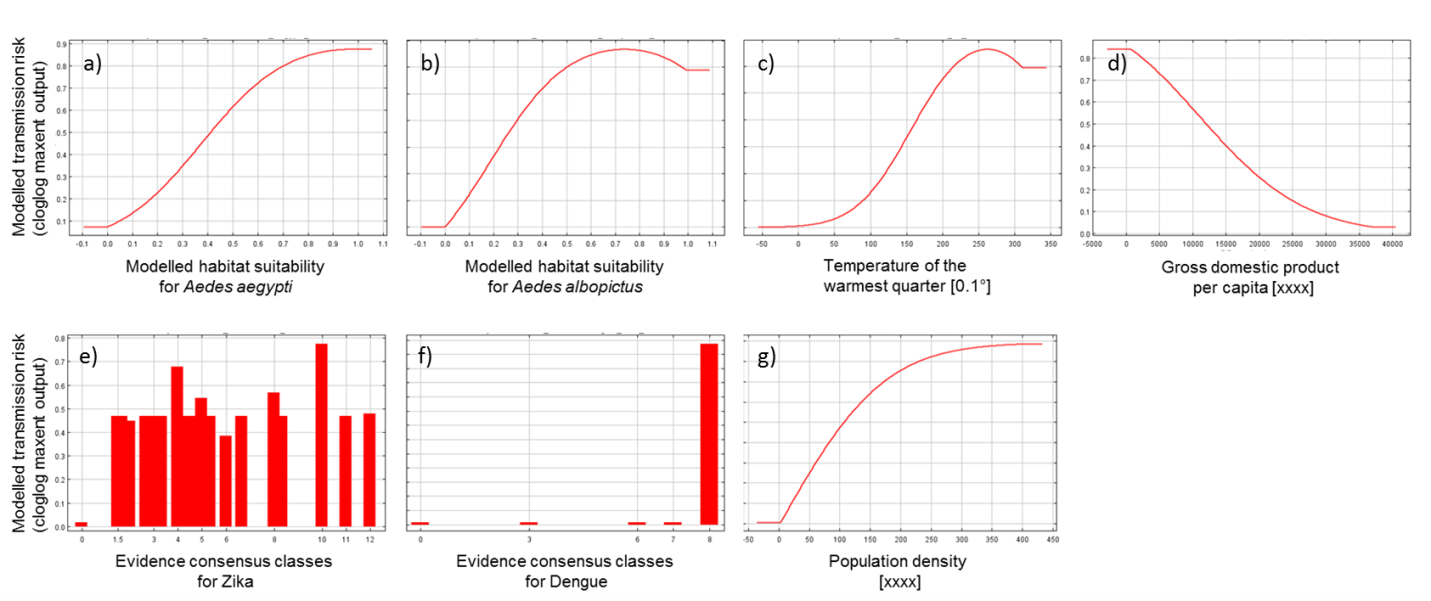


**Figure S1:** One-variable response curve for the seven variables tested as explanatory variables for the geographical distribution of the ZIKV transmission risk in South and Central America.


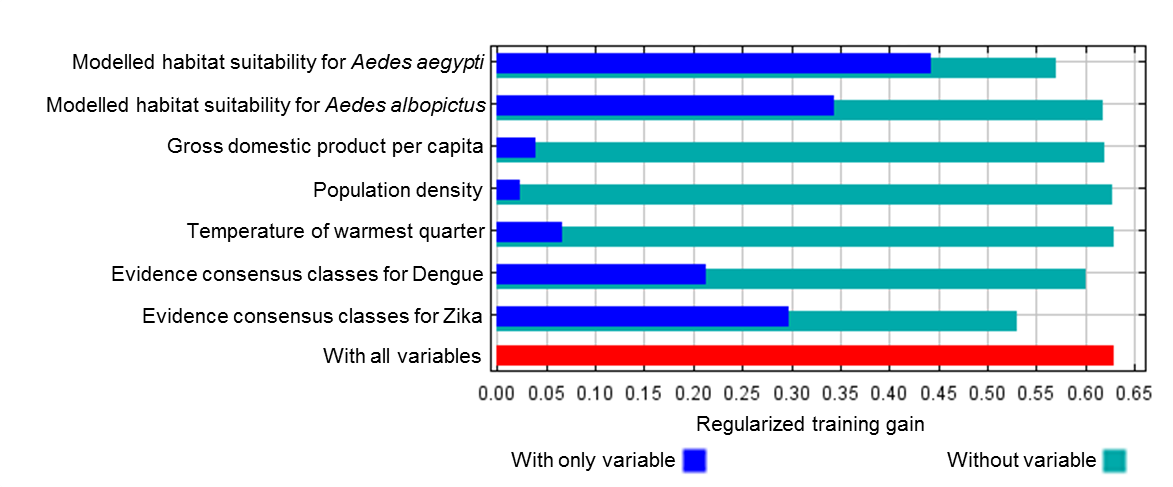


**Figure S2:** Results of the jack-knifing test for variable importance implemented in Maxent. Note the scale from 0-0.65 variable importance.

Figure S3: Occurrence records used for modelling the habitat suitability of the vector distribution. a) Occurrence records for *Aedes aegypti* and b) occurrence records for *Aedes albopictus*. Data taken from Kraemer et al., 2015a,b.

Figure S4: Potential vector distribution and temperature conditions suitable for ZIKV transmission. Areas in red are modelled to be suitable for at least one of the two main vectors species *Aedes aegypti* and *Aedes albopictus*. Hatched areas match the respective temperature criteria suitable for the ZIKV provided by Mordecai et al. (2017) regarding the mean temperature of warmest quarter.
